# Digital Self-Management Intervention Paths for Early Breast Cancer Patients: Results of a Pilot Study

**DOI:** 10.1155/2024/8036696

**Published:** 2024-08-13

**Authors:** Paula Poikonen-Saksela, Evangelos Karademas, Leena Vehmanen, Meri Utriainen, Haridimos Kondylakis, Konstadina Kourou, Georgios C. Manikis, Eleni Kolokotroni, Panagiotis Argyropaidas, Berta Sousa, Ruth Pat Horenczyk, Ketti Mazzocco, Johanna Mattson

**Affiliations:** ^1^ Comprehensive Cancer Center Helsinki University Hospital and University of Helsinki, Helsinki, Finland; ^2^ Department of Psychology University of Crete, Rethymno, Greece; ^3^ Foundation for Research and Technology-Hellas Institute of Computer Science, Heraklion, Greece; ^4^ Department of Material Science and Engineering University of Ionnina, Ionnina, Greece; ^5^ Foundation for Research and Technology-Hellas Biomedical Research Institute, Ionnina, Greece; ^6^ In Silico Oncology and In Silico Medicine Group Institute of Communication and Computer Systems School of Electrical and Computer Engineering National Technical University of Athens, Athens, Greece; ^7^ Breast Unit Champalimaud Clinical Centre/Champalimaud Foundation, Lisboa, Portugal; ^8^ School of Social Work and Social Welfare The Hebrew University of Jerusalem, Jerusalem, Israel; ^9^ Applied Research Division for Cognitive and Psychological Science European Institute of Oncology IRCCS, Milan, Italy; ^10^ Department of Oncology and Hemato-Oncology University of Milan, Milan, Italy

## Abstract

**Background:**

Despite excellent prognosis of early breast cancer, the patients face problems related to decreased quality of life and mental health. There is a need for easily available interventions targeting modifiable factors related to these problems. The aim of this study was to test the use of a new digital supportive intervention platform for early breast cancer patients. *Material and Methods*. Ninety-seven early breast cancer patients answered questions on wellbeing, exercise, and sociodemographic factors before systemic adjuvant treatment at the Helsinki University Hospital. Based on these answers and predictive algorithms for anxiety and depression, they were guided onto one or several digital intervention paths. Patients under 56 years of age were guided onto a nutrition path, those who exercised less than the current guideline recommendations onto an exercise path, and those at risk of mental health deterioration onto an empowerment path. Information on compliance was collected at 3 months on the amount of exercise and quality of life using EORTC-C30 scale, anxiety and depression using HADS scale at baseline and 12 months, and log-in information at 3 and 12 months.

**Results:**

Thirty-two patients followed the empowerment path, 43 the nutrition path, and 75 the exercise path. On a scale of 1–5, most of the participants (mean = 3.4; SD 0.815) found the interventions helpful and would have recommended testing and supportive interventions to their peers (mean = 3.70; SD 0.961). During the 10-week intervention period, the mean number of log-ins to the empowerment path was 3.69 (SD = 4.24); the nutrition path, 4.32 (SD = 2.891); and the exercise path, 8.33 (SD = 6.293). The higher number of log-ins to the empowerment (rho = 0.531, *P*=0.008, and *n* = 24) and exercise paths (rho = 0.330, *P*=0.01, and *n* = 59) was related to better global quality of life at one year. The number of log-ins correlated to the weekly amount of exercise in the exercise path (cc 0.740, *P* value <0.001, and *n* = 20).

**Conclusion:**

Patients' attitudes towards the interventions were positive, but they used them far less than was recommended. A randomized trial would be needed to test the effect of interventions on patients' QoL and mental health.

## 1. Introduction

Breast cancer is the most common cancer in the world, but modern treatment has made its prognosis excellent [1]. In early breast cancer, quality of life (QoL) is often impaired during active treatment due to side-effects, but it usually starts to improve within the first years [2]. However, QoL can remain impaired for several years after the diagnosis [3]. It is important to find modifiable targets for interventions to promote QoL and wellbeing of patients with early breast cancer. Exercise is known to have many positive effects on breast cancer patients' wellbeing [4, 5]. Exercise interventions or physical activity in general have positive effects on quality of life (QoL) [6], fatigue [7], and physical fitness [4]. There is also evidence of an inverse association between physical activity and exercise after breast cancer diagnosis and breast cancer and/or all-cause mortality [8]. The European Society for Medical Oncology (ESMO) clinical practice guidelines and several clinical breast cancer studies claim that addressing patients' long-term psychological needs can promote their QoL and overall wellbeing [9]. The psychological features and symptoms of early breast cancer patients are predictors of long-term mental health deterioration [10]. Most women gain weight after breast cancer treatment. Weight gain has a negative effect on QoL, especially among premenopausal breast cancer patients, who often develop chemotherapy-induced premature menopause [11]. Exercise, nutrition, and mental health are logical targets for supportive interventions among early breast cancer patients [6, 11, 12].

According to a recent meta-analysis [13], electronic health (eHealth) offers promising means to improve the overall QoL of women with breast cancer via the internet and related technologies (websites, software, etc.). In this meta-analysis, eHealth interventions for breast cancer patients were associated with statistically significant improvements in QoL compared with usual care, regardless of the participants' characteristics, features of the eHealth platforms, timing of the interventions, or assessment scales [13]. eHealth services are also available for a broad spectrum of patients.

The BOUNCE project (Predicting Effective Adaptation to Breast Cancer to Help Women to BOUNCE Back–the European Union's Horizon 2020 research and innovation programme under grant agreement no. 777167) studied the resilience of early breast cancer patients and developed algorithms to predict their mental health during adjuvant therapy [10, 14]. In line with the BOUNCE project, we considered psychological symptoms, exercise habits, and nutrition as possible targets for supportive interventions among patients with early breast cancer. Our study aimed to test the use of a digital supportive intervention palette containing information and self-management techniques for psychological empowerment, exercise, and nutrition for early breast cancer patients on the Helsinki University Hospital's (HUS) Health Village platform. Another aim of the study was to examine the value of the targeted digital intervention from the patient perspective (meaningfulness, recommendability, and preference), the self-reported use of these digital interventions, the patients' log-in information, and the preliminary effect of digital intervention paths on exercise habits (exercise path), general QoL, and mental health (all paths). The novelty and importance of this study lies in the fact that it was based on the findings of a previous large-scale study regarding adaptation to breast cancer (i.e., BOUNCE) and that it used a new digital supportive intervention platform.

## 2. Materials and Methods

### 2.1. Study Participants

The BOUNCE project studied the resilience of early breast cancer patients. Between December 2020 and September 2021, 97 participants were recruited from HUS for the BOUNCE intervention study before beginning their oncological systemic treatment. This was a substudy with a separate patient population to that of the main BOUNCE study to predict resilience. The participants had been diagnosed with stage I–III breast cancer and were to receive some type of systemic treatment in an adjuvant or neoadjuvant setting. They had no early onset, severe psychiatric disease, or other major illness or operation in the last year. The inclusion and exclusion criteria and the data collection procedure are described in detail elsewhere [15]. For this intervention study, the data to evaluate the need for interventions were collected using the Noona tool [16] and patients had to be willing to use a digital platform for the interventions. The data were exported from the Noona tool to a separate Integrated Clinical Decision Support Tool incorporating predictive algorithms (able to predict mental health) [17] and an exercise calculator (recommending more exercise to patients who exercised less than recommended).

### 2.2. Digital Intervention

**Figure 1:**
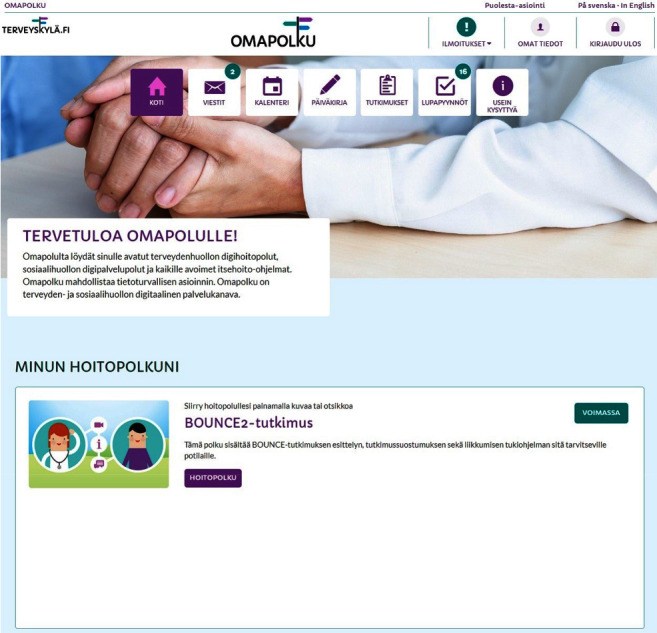
Interface for BOUNCE intervention path in HUS Health Village (Terveyskylä).

The digital platform for the interventions to support resilience after breast cancer diagnosis was designed by professionals (oncologists, physiotherapists, nutrition therapists, and psychologists) with experience in supportive interventions for breast cancer patients. The platform consisted of three supportive paths, namely, the exercise path, the empowerment path, and the nutrition path. The active intervention period lasted 10 weeks, and the material was available to the patients for up to one year. At the baseline, a trial assistant introduced the HUS Health Village platform with the BOUNCE intervention paths to the participants (Figure 1), either face-to-face or by phone. The participants were allowed to contact the trial assistant if they had any technical problems. The research team selected the intervention paths on the basis of the following criteria: (a) exercise path: participants who exercised (moderate exercise) less than 150 minutes per week and/or did muscle training less than twice a week; (b) empowerment path: participants at a high risk of anxiety or depression during the following three- to six-month period, according to the BOUNCE predictive models [14]; and (c) nutrition path: patients aged under 56 and at risk of weight gain after breast cancer treatments due to premature menopause. The patients were able to take part in several paths simultaneously. Information on the participants' compliance was collected at 3 months (after the 10-week intervention period); information on the participants' amount of exercise (in the exercise path) and data on QoL, anxiety, and depression were collected at baseline and 12 months after recruitment; and log-in information was collected from the platform 3 (after the 10 weeks intervention period) and 12 months after recruitment.

On the exercise path, we encouraged the participants to exercise according to current recommendations, at least 150 minutes of moderate aerobic exercise per week and muscle training twice a week, their condition permitting. They received information on appropriate exercise for cancer patients. We also asked the participants to exercise at least once a week using a recorded online session on the My Path platform (https://www.terveyskyla/omapolku). These circuit training sessions were planned by physiotherapists of the HUS Comprehensive Cancer Center. One session lasted 33–38 minutes; a lighter and heavier version, as well as a stretching video, was also available. Every six weeks, the participants joined an online group chat to receive more personal guidance and discuss their questions about exercise. Walks in nature with a group of peers and a physiotherapist were also offered every one to two months. The participants kept a digital diary on the amount and type of exercise they did.

If at least one of the four BOUNCE predictive mental health algorithms [14] showed a risk of symptoms of anxiety or depression in the next three to six months, the participant was encouraged to use the HUS patient empowerment platform in the digital Health Village's My Path (https://www.terveyskyla/omapolku). Participants with severe psychosocial problems (Hospital Anxiety and Depression Scale (HADS) score for anxiety or depression of 13 or more at the baseline or at follow-up) were offered an appointment at the psychosocial unit, in accordance with local clinical practice. The empowerment path consisted of psychoeducation on the following different themes: anxiety, relaxation, coping with everyday life, worry, being present, thoughts and beliefs, self-compassion, and strength. We asked the participants to use the relevant supportive module two to three times a week during the first 10 weeks and to continue according to their own preferences after this. The empowerment module did not involve any direct contact with the psychosocial or care team.

The nutrition intervention path was offered to participants aged 40–55, who were at risk of weight gain due to early menopause caused by breast cancer therapies. They received information and videos about the effect of weight gain on prognosis, QoL, and the wellbeing of breast cancer patients. The nutrition path also included digital tasks for a period of 10 weeks and a monthly group chat. Digital tasks were practiced to detect eating habits, content of meals, hunger and satiety, and desires, as well as strength and motivation for changes.

## 3. Outcome Assessment

We collected all the log-in information from the three paths. During the follow-up, QoL was measured using the global health status/QoL scale of the EORTC QLQ-C30 (European Organization for the Research and Treatment of Cancer Quality of Life Questionnaire) [18], which assess patients' subjective QoL and is based on two questions about perceived physical condition and overall QoL [18]. We used HADS to assess the psychological symptoms of anxiety and depression [19]. Exercise was calculated in minutes per week. The specific compliance questionnaire was developed for this study to assess participants' overall evaluation of the digital intervention and is presented in Table 1. It includes seven simple questions asking participants to evaluate the usefulness, helpfulness, ease of use, and attractiveness of the intervention.

### 3.1. Statistical Methods

Due to the relatively small number of participants and the non-normally distributed data in the exercise-related variables (Kolmogorov–Smirnov *Z* > 1.40, *P* < 0.05), we used the following nonparametric statistical analyses: Spearman rho correlations to examine the associations between the variables and the Wilcoxon *Z* to measure the potential changes in the amount of time (in minutes) that the participants allocated to physical exercise per week, from the baseline to follow-up at 12 months. We also carried out a chi-square test to examine the potential changes in the physical exercise-related recommended activity (i.e., at least 150 min of moderate exercise per week) from the baseline to 12-month follow-up. Finally, frequencies (e.g., means and percentages) were reported.

## 4. Results

Table 2 presents the characteristics of the 97 participants at the beginning of the systemic therapy. Thirty-two participants enrolled (not exclusively) on the empowerment path, 43 participants on the nutrition path, and 75 on the exercise path. Forty (41.24%) people enrolled on only the exercise path, 19 (19.59%) on all paths, 25 (25.77%) on both the exercise and nutrition path, and 13 (13.40%) enrolled on both the exercise and empowerment path.

After the intervention period, on a scale of 1–5, most of the participants considered the interventions helpful (mean = 3.4; SD = 0.815) and would have recommended having their wellbeing tested to others (mean = 3.70; SD = 0.961). Twenty patients (20.6%) would have preferred some other type of personal support, and 36 (37.1%) felt that they would have managed without any support at all.

Two participants (2.1%) reported that they had used the intervention paths as recommended, 23 participants (23.7%) reported that they had used the intervention paths for 50–80% of the recommended time, 35 (36.1%) had used the intervention paths occasionally, and 17 (17.5%) had not used the interventions at all. Table 1 presents the responses to the compliance questionnaire.

The mean number of log-ins during the intervention period presented in Table 3 varied from 3.69 (range = 1–17) for the empowerment path to 8.33 (range = 1–34) for the exercise path. The mean number of log-ins during the one-year follow-up varied from 4.86 (range = 1–21) for empowerment path to 14.82 (range = 1–34) for exercise path. The mean number of all log-ins to any of the paths during the year was 18.11 (range = 2–51) for the whole group and 19.1 (range = 2–51) for the exercise group.

The higher number of log-ins during the intervention period was related to better global QoL at 12 months on the exercise (Spearman rho = 0.330, *P*=0.01, and *n* = 59) and the empowerment path (rho = 0.531, *P*=0.008, and *n* = 24). The number of log-ins correlated with the mean exercise time reported (in a relevant diary) by the participants on the exercise path (rho = 0.740, *P* < 0.001, and *n* = 20). We found no significant correlation between the number of log-ins and anxiety or depression on the exercise or the empowerment path, nor between the number of log-ins and QoL or anxiety or depression on the nutrition path. The amount of exercise increased after the exercise intervention from *M* = 220.16 (SD = 107.61) minutes at the baseline to *M* = 230.07 (SD = 114.93) minutes after one year, but this change was not statistically significant (Wilcoxon *Z* = −0.56, *P*=0.211, *n* = 68).

Moreover, of the 19 participants who were enrolled on the exercise path and were “inactive” (according to the recommendation of at least 150 min of moderate exercise per week) at the baseline, 10 (52.6%) had become “active” at 12 months (vs 9 (47.4%) who remained “inactive”), whereas of the 49 “active” participants at the baseline, only seven (14.3%) had become “inactive” at 12 months (chi-square = 8.33, *P* < 0.01).

## 5. Discussion

According to a recent meta-analysis, eHealth is superior to usual care for women with breast cancer in terms of improved QoL and is a useful tool for enhancing health services and collecting patient information via the internet [13].

The BOUNCE project focused on predicting the levels of resilience of early breast cancer patients based on a wide battery of biomedical and psychosocial variables collected mainly using the Noona platform [15]. In this study, we tested the first BOUNCE predictive models for anxiety and depression as resilience-related outcomes among early breast cancer patients. The main purpose of this pilot study was to test the concept of using predictive tools to target digital interventions and especially to gain patients' perspectives of the process.

The intervention platform used in this study was designed for the HUS Health Village platform. It was based on information and self-management and offered little personal contact via chat or group meetings (walking in nature).

In general, the participants felt positive about their resilience being measured and about the targeted interventions. They also felt that the interventions were helpful. Only 20% of them would have preferred another type of contact, for example, a face-to-face meeting during the study period, but as the study was conducted during the COVID-19 pandemic, this was not always possible. Thirty-seven per cent of the participants felt they would have managed without the interventions, which shows that our criteria for the interventions were quite inclusive and that the selected participants already had good subjective QoL. The fact that the participants already felt good could partly explain why they used the intervention portal less than recommended. The same patient could also take several paths, which may have required too much effort during the busy early period of oncological treatments. A more suitable time point for actively using digital interventions could be after the busiest treatment period. However, information on the patients' wellbeing from the very beginning of treatments would be valuable. In the literature, breast cancer patients' engagement in eHealth interventions varies, with 0–100% of users completing various intervention modules [20]. Engagement in eHealth interventions decreases over time and interactive support seems to be the most engaging [20]. Our results were in line with this; the participants on the exercise path, who were offered both the chat and group meetings (walking in nature) were more active users than those on the other paths. The activity of those on the empowerment path, with no contact with others, was lower than that of those on the other paths.

Most of the participants (*n* = 75) were guided onto the exercise path. The main reason for this was that 66% of the participants did not meet the criteria of muscle training twice a week recommended in the exercise guidelines for cancer patients [21]. Considering their approximately 220 mean minutes of moderate exercise at the baseline, they were quite active already, and the intervention may have helped them remain so, as exercise minutes had slightly increased to about 230 after one year. Unfortunately, muscle training was reported as too inactive throughout the pilot study to be able to make meaningful comparisons. Recently, two meta-analyses revealed that various eHealth interventions, also focusing on physical activity, had a significant effect on QoL but no effect on anxiety or depression [13, 20]. Although active use of the exercise path seemed to be related to better QoL in our pilot study, no strong conclusion can be drawn about causality due to the nonrandomised study design.

The novelty of this pilot study is that we used a recently designed digital multicomponent self-management platform as well as an early version of a predictive tool to choose the patients for each intervention path.

However, the study also had some limitations, namely, that its design may have led us to specifically select those who were interested in their wellbeing and thus used our paths inactively as they felt no need for such support. We also used a nonvalidated compliance questionnaire, which was developed for this study targeting many aspects of the process in one short questionnaire.

### 5.1. Conclusion

The participants' attitudes towards the interventions were positive, but they used them less than recommended. The participants on the exercise path were more active than the others. Nevertheless, more research and a randomised trial on the effect of interventions on patients' QoL and mental health are warranted.

## Figures and Tables

**Table 1:** Responses to compliance questionnaire three months after intervention period (*n* = 97).

Question	1	2	3	4	5	Nonrespondents
Did you find it effortless to answer the questions on noona to determine your resilience? (1 = I completely disagree, 5 = I completely agree)	2 (2.1)	4 (4.1)	26 (26.8)	28 (28.9)	17 (17.5)	20 (20.6)
Do you feel that measuring your resilience was useful?	1 (1)	6 (6.2)	38 (39.2)	25 (25.8)	7 (7.2)	20 (20.6)
Do you feel that the targeted interventions were helpful?	2 (2.1)	11 (11.3)	31 (32)	21 (21.6)	12 (12.4)	20 (20.6)
Would you recommend measuring resilience and targeted digital interventions to other breast cancer patients?		2 (2.1)	16 (16.5)	27 (27.8)	32 (33)	20 (20.6)
Have you used any digital interventions in the last three months?	17 (17.5)	35 (36.1)	10 (10.3)	13 (13.4)	2 (2.1)	20 (20.6)
Would you have preferred other types of intervention than digital, e.g., face-to-face meetings?	Yes 20 (20.6)	No 57 (58.8)				20 (20.6)
Would you have managed without any support?	Yes 36 (37.1)	No 41 (42.3)				20 (20.6)

Scale for questions 1 to 4 : 1 = I completely disagree, 5 = I completely agree; for question 5 : 1 = not at all, 2 = occasionally, 3 = 50% of the recommended time, 4 = 50–80% of the recommended time, 5 = at least 80% of the recommended time.

**Table 2:** Patient characteristics.

Variable	Mean (SD)	Range
Age, years *n* = 96	58	41–91
BMI, kg/m^2^*n* = 91	26.66	17.72–40.61
Baseline general QoL *n* = 95	71.49	16.67–100
Baseline HADS anxiety *n* = 96	0.92 (0.49)	0–3
Baseline HADS depression *n* = 96	0.49 (0.46)	0–2.29
Baseline HADS total	0.71 (0.42)	0–2.36

	Number of participants	% Of patients (*n* = 97)

ECOG *n* = 97		
0	77	79.4%
1	17	17.5%
** **2	3	3.1%
Education *n* = 96		
0–9 years	13	13.4
>9 years	83	85.6
Marital status *n* = 96		
Married/cohabiting	75	77.3
Single/widowed	21	21.6
Working *n* = 96		
Employed	82	84.5
Nonemployed/retired	14	14.4
Income *n* = 93		
Very low/low	15	15.5
Average/high	78	80.4
Tumour grade *n* = 95		
I	19	57.9
II	44	37.9
III	32	4.2
Cancer stage *n* = 97		
I	55	56.7
II	36	37.1
III	4	4.1
Radiotherapy *n* = 97		
Yes	87	82.5
No	10	16.5
Endocrine treatment *n* = 97		
Yes	88	90.7
No	9	9.3
Chemotherapy *n* = 97		
Yes	52	53.6
No	45	46.4
AntiHER2 treatment *n* = 96		
Yes	16	82.5
No	80	16.5

**Table 3:** Log-in information of intervention paths.

Path	Log-ins during intervention period mean (SD; range)	Log-ins over one year mean (SD; range)	Path completed *n* (%)	No log-ins at all *n* (%)
Exercise (*n* = 75)	8.42 (6.27; 1–27)	15.01 (8.49; 1–34)	31 (41.3%)	0
Nutrition (*n* = 43)	4.32 (2.89; 0–12)	7.46 (5.79; 1–25)	3 (6.98%)	3 (6.98%)
Empowerment (*n* = 32)	3.69 (4.24; 0–17)	4.86 (5.16; 1–21)	3 (9.4%)	3 (9.38%)
All paths (*n* = 97)	8.79 (9.07; 1–43)	18.33 (12.28; 2–51)	32 (33%; at least one path)	0

## Data Availability

The data that support the findings of this study are available on request from the corresponding author. The data are not publicly available due to restrictions, for example, they contain information that could compromise the privacy of the study participants.
